# Food safety in hospital: knowledge, attitudes and practices of nursing staff of two hospitals in Sicily, Italy

**DOI:** 10.1186/1472-6963-7-45

**Published:** 2007-04-03

**Authors:** Cecilia Buccheri, Alessandra Casuccio, Santo Giammanco, Marco Giammanco, Maurizio La Guardia, Caterina Mammina

**Affiliations:** 1 Division of Physiology and Human Nutrition, Department of Medicine, Pneumology, Physiology and Human Nutrition, University, Via A. Elia, 90127 Palermo, Italy; 2 Department of Clinical Neurosciences, University, Via L. Giuffrè, 90127 Palermo, Italy; 3 Department of Hygiene and Microbiology "G. D'Alessandro", University, Via del Vespro 133, 90127 Palermo, Italy

## Abstract

**Background:**

Food hygiene in hospital poses peculiar problems, particularly given the presence of patients who could be more vulnerable than healthy subjects to microbiological and nutritional risks. Moreover, in nosocomial outbreaks of infectious intestinal disease, the mortality risk has been proved to be significantly higher than the community outbreaks and highest for foodborne outbreaks. On the other hand, the common involvement in the role of food handlers of nurses or domestic staff, not specifically trained about food hygiene and HACCP, may represent a further cause of concern.

The purpose of this study was to evaluate knowledge, attitudes, and practices concerning food safety of the nursing staff of two hospitals in Palermo, Italy. Association with some demographic and work-related determinants was also investigated.

**Methods:**

The survey was conducted, by using a semi-structured questionnaire, in March-November 2005 in an acute general hospital and a paediatric hospital, where nursing staff is routinely involved in food service functions.

**Results:**

Overall, 401 nurses (279, 37.1%, of the General Hospital and 122, 53.5%, of the Paediatric Hospital, respectively) answered. Among the respondents there was a generalized lack of knowledge about etiologic agents and food vehicles associated to foodborne diseases and proper temperatures of storage of hot and cold ready to eat foods. A general positive attitude towards temperature control and using clothing and gloves, when handling food, was shared by the respondents nurses, but questions about cross-contamination, refreezing and handling unwrapped food with cuts or abrasions on hands were frequently answered incorrectly. The practice section performed better, though sharing of utensils for raw and uncooked foods and thawing of frozen foods at room temperatures proved to be widely frequent among the respondents. Age, gender, educational level and length of service were inconsistently associated with the answer pattern.

More than 80% of the respondent nurses did not attend any educational course on food hygiene. Those who attended at least one training course fared significantly better about some knowledge issues, but no difference was detected in both the attitude and practice sections.

**Conclusion:**

Results strongly emphasize the need for a safer management of catering in the hospitals, where non professional food handlers, like nursing or domestic staff, are involved in food service functions.

## Background

In the last decades, the epidemiology of foodborne diseases is changing with new or unexpected pathogens often emerging on a countrywide or worldwide scale, new foods expanding the range of potential vehicles of pathogens, wider social contexts being involved and new classes of individual being at higher risk [1,2]. These changes may be attributable to several socio-economic and demographic factors, including dramatic quali-quantitative changes in primary production, processing, distribution and handling of food and the increasing exposure of individuals, like elderly, patients with impaired immunity and many hospitalized subject [3-6].

Epidemiological and surveillance data suggest that faulty practices in food processing plants, food service establishments and home play a crucial role in the causal chain of foodborne diseases. This issue has also proved to be critical in some nosocomial foodborne outbreaks [7-11]. Hence, a major goal of the hospital is to provide safe food to patients who frequently are at higher risk of acquiring infections and their complications [12,13].

In Italy, like in other European countries, great efforts are being put in place to promote food safety at all levels of the food chain, and the European legislation has mandated that all food businesses adhere to the Hazard Analysis and Critical Control Points (HACCP) system [14]. The importance of food hygiene training is also experiencing a growing acknowledgement by both food workers and the official control systems in Europe. Both the European Directive 93/43 and the more recent Regulation 852/2004 require food hygiene training for every food handler [15].

Food hygiene in the hospital can acquire peculiar features: indeed, many patients could be more vulnerable than healthy subjects to microbiological and nutritional risks; large numbers of persons can be exposed to infections and possible complications; gastroenteritis can impair digestion and absorption of nutrients and the perception or fear about poor food hygiene practices might result in patients rejecting the meals supplied by the hospital catering [12,13]. In nosocomial outbreaks of infectious intestinal disease, the mortality risk has been proved to be significantly higher than the community outbreaks and highest for foodborne outbreaks [16]. On the other hand, further peculiar concern arises from the common involvement in the role of food handlers of nurses or domestic staff, who are not specifically trained about food hygiene and HACCP, but can be engaged in receipt, distribution and serving of ready made foods and supervision of these services.

The aim of this study was to evaluate knowledge, attitudes and practices of the nurses routinely involved in food service functions in two hospitals of Palermo, Italy. Association with some demographic and work-related variables was also investigated.

## Methods

### Setting

For the purpose of the study, we selected two hospitals included into the Azienda di Rilievo Nazionale ed Alta Specializzazione (ARNAS) in Palermo, Italy: a) the acute general hospital "Civico and M. Ascoli" with 43 wards and 900 beds; b) the paediatric hospital "G. Di Cristina" with 13 wards and 220 beds.

The two hospitals are contracted out to an external caterer under the regulations in force in Italy and the additional conditions specified by the hospital board. The catering service is provided by a single premise, where an HACCP plan is in place since 2000 and a quality assurance program is running in accordance with the UNI EN ISO 9002 standards. The meals are plated individually according to patient needs and wants, stored and transported in either a heated or cooled state and delivered to wards for immediate distribution. On arrival at the ward level, a random checking to ensure that food is served at the correct temperature is performed by the nursing staff. This is charged also with the function of monitoring the intervals of time between reception and distribution of meals and the hygienic practices of the domestic staff through the entire food handling procedure the until disposal of wastes. To ensure that correct qualitative standards are being adhered to, a monitoring program is in place by the medical direction of the two hospitals to assess the quality of food, hygiene, production standards, specifications and service.

### Survey instrument

From March to November 2005, knowledge, attitudes and practices about food hygiene of the nursing staff of the two hospitals under study were investigated by means of a questionnaire survey. The medical directors of the hospitals were sent a letter explaining the purpose and the investigation procedure. Because the survey data did not influence patient management and the issue being investigated is a matter of public record, ethical approval for the study was not required. After obtaining approval, the questionnaire was addressed to all nurses potentially involved in food related functions in the acute general hospital "Civico and M. Ascoli" and the paediatric hospital "G. Di Cristina" (751 and 228, respectively). To overcome the difficulties arising from the habitual 8-hours shifts of the health care workers and minimize the non-respondent prevalence, the questionnaire was self-administered. Confidentiality of the answers was also warranted.

A questionnaire (see the additional file 1), containing mainly multiple-choices questions, was prepared based upon questionnaires previously used in studies done in Italy and in other countries [17-20]. It included five sections: a) demographic characteristics, employment status and hospital/ward where the nurse worked; b) knowledge about food hygiene; c) attitudes towards prevention of foodborne diseases; d) measures to be used in prevention of foodborne diseases; e) sources of information about food hygiene. The questions concerning knowledge about foodborne disease agents and foods epidemiologically linked to transmission of pathogens listed some microrganisms, and respondents were asked to choose from among three options – yes, no, don't know – about their association with foodborne disease and to couple at least one food vehicle to each pathogen. A question on a non-foodborne agent of special interest for healthcare workers, hepatitis B virus (HBV), was added. Answers were classified as correct when they contained exclusively food items whit a well recognized role as a vehicle for the pathogen under analysis. The answers in the section of practices were simplified, including only three choices (always, often, occasionally).

### Statistical procedures

Food safety knowledge, attitude and practice scores for the respondents were, respectively, calculated based on the multiple choice answer to each statement, only for the items where the proportion of correct answers was 95% or less. It was assigned a score: +1 when the correct option had been checked off, -1 in the case of incorrect option and 0 in the case of don't know/uncertain option. The total percent score for the respondents nurses was then calculated by dividing the score sum by the maximum possible score.

Statistical analysis of association between questionnaire answers and demographic and work-related characteristics, such as hospital and length of service in the employment/ward, was performed by categorization of answers for each section as dichotomous variables: knowledge was categorized and recorded as correct vs. incorrect/unknown, attitudes as agreement vs. disagreement/uncertain and practices as safe, when answer was always (occasionally for the question D6), vs. unsafe, when answer was often or never (always for the question D6).

Data were analyzed by the Epi Info software (version 6.0, CDC, Atlanta, GA, US) and the SPSS Software 14.0 version (SPSS, Inc., Chicago, Ill, US). The one-way analysis of variance (ANOVA) was used to evaluate difference in parametric variables. Frequency analysis was performed with chi-square (χ^2^) test. Cross-tabulation and χ^2 ^tests were carried out to determine the relationship between nurses' knowledge, attitudes and practices and demographic and work-related data. Moreover, to explore whether this relationship systematically varied by specific sociodemographic characteristics, a series of logistic regression analyses were conducted. Independent variables included age, gender, education, length of service in the employment/ward and having attended courses on food hygiene. In all analyses, differences were considered statistically significant at *P *≤ 0.05.

## Results

Of the nurses working, respectively, in the general hospital "Civico and Maurizio Ascoli" and the paediatric hospital "G. Di Cristina", 37.1% (279/751) and 53.5% (122/228), respectively, returned the compiled questionnaire. Table 1 provides a description of the respondents in the study and the differences between the demographic characteristics of the nursing staff of the two hospitals under study. The majority (60.6%) were female. Age of the respondents ranged between 24 and 64 years. Mean age and length of service as a nurse did not significantly differ between the two hospitals. Length of service in the ward where the respondents worked at the time of investigation significantly differed as a mean of years, but when stratified on a ten-year basis did not. Moreover, the most frequently reported education level was a high school diploma (13 years). A statistically significant difference in the education level was noticed between the respondents of the two hospitals.

**Table 1:** Demographic characteristics of survey respondents among the nursing staff of the two hospitals

Characteristics of survey respondents	General	Paediatric	P
Gender			
Male	114 (40.9)	44 (36.1)	0.346
Female	165 (59.1)	78 (63.9)	
Total number	279 (100.0)	122 (100.0)	
			
Age (yr)			
<35	61 (22.6)	20 (17.4)	0.215
35–50	162 (60.0)	67 (58.3)	
>50	47 (17.4)	28 (24.3)	
Total number	270 (100.0)	105 (100.0)	
Mean (range)	42.0 (24–64)	44.0 (31–64)	0.054
			
Length of service in the employment (yr)			
≤10	60 (23.2)	21 (18.1)	0.166
11–20	109 (42.1)	43 (37.1)	
≥21	90 (34.7)	52 (44.8)	
Total number	259 (100.0)	116(100.0)	
Mean (range)	18.0 (0–39)	20.0 (3–39)	0.063
			
Length of service in the present ward (yr)			
≤10	77 (31.4)	26 (22.6)	0.067
11–20	96 (39.2)	42 (36.5)	
≥21	72 (29.4)	47 (40.9)	
Total number	245 (100.0)	115 (100.0)	
Mean (range)	16.0 (0–37)	18.0 (3–36)	0.025*
			
Education level (yr)			
8	41 (14.7)	28 (22.9)	0.012*
13	171 (61.3)	80 (65.6)	
≥16	14 (5.0)	0	
Total number	226 (100.0)	108 (100.0)	
			
Attending at least one course on food hygiene and foodborne diseases in hospital			
No	218 (78.1)	107 (87.7)	0.057
Yes	32 (11.5)	7 (5.7)	
Unanswered	29 (10.4)	8 (5.7)	
Total number	279 (100.0)	122 (100.0)	

* statistical significance

Tables 2 to 4 summarize the most significant findings obtained by the administration of the questionnaire with reference to the sections of knowledge, attitudes and practices, respectively. Only the answers where agreement within respondents was 95% or lower were considered.

**Table 2:** Respondent's food safety knowledge

Statement	Responses *n *(%)				Score
	Correct	Not correct	Don't know	Unanswered	
**B1. Preparation of food in advance is likely to contribute to food poisoning**	273 (68.1)	92 (23.0)	29 (7.2)	7 (1.7)	181
**B2. Reheating of food is likely to contribute to food contamination**	367 (91.5)	17 (4.2)	16 (4.0)	1 (0.3)	350
**B6. Wearing gloves while handling food minimizes risk of transmitting infection to food-service staff**	245 (61.1)	124 (30.9)	22 (5.5)	10 (2.5)	121
**B7. The correct temperature for a refrigerator is (°C)**	269 (67.1)	113 (28.2)	/	19 (4.7)	156
**B8. Hot ready to eat foods should be maintained at (°C)**	17 (4.2)	335 (83.5)	/	49 (12.3)	-318
**B9. Cold ready to eat foods should be maintained at (°C)**	230 (57.4)	151 (37.7)	/	20 (4.9)	79
**B10b. Hepatitis B can be transmitted by food**	153 (38.2)	35 (8.7)	7 (1.7)	206 (51.4)	118
**B10d. Cholera can be transmitted by food**	271 (67.6)	45 (11.2)	12 (3.0)	73 (18.2)	226
**B11d. Food items associated to the transmission of *Vibrio cholerae***	119 (29.7)	24 (6.0)	/	258 (64.3)	95
**B11f. Food items associated to the transmission of gastroenteritis**	109 (27.2)	213 (53.1)	/	79 (19.7)	-104
	TOTAL SCORE/FULL SCORE (%)				904/3288 (27.5)

**Table 3:** Respondent's food safety attitudes

Statement	Responses *n *(%)				Score
	Yes	No	Uncertain	Unanswered	
**C1. Raw foods should be kept separated from cooked foods**	314 (78.3)	64 (16.0)	13 (3.2)	10 (2.5)	250
**C2. Defrosted food should not be refrozen**	45 (11.2)	348 (86.8)	2 (0.5)	6 (1.5)	-303
**C7. Food-service staff with abrasions or cuts on hands should not touch unwrapped food**	340 (84.8)	51 (12.7)	3 (0.8)	7 (1.7)	289
	TOTAL SCORE/FULL SCORE (%)				236/1180 (20.0)

**Table 4:** Respondent's food safety practices

Statement	Responses *n *(%)				Score
	Always	Often	Occasionally	Unanswered	
**D1. Do you wash your hands before touching unwrapped raw food?**	314 (78.3)	77 (19.2)	6 (1.5)	4 (1.0)	308
**D2. Do you wash your hands after touching unwrapped raw food?**	315 (78.6)	78 (19.4)	4 (1.0)	4 (1.0)	311
**D3. Do you wash your hands before touching unwrapped cooked food?**	335 (83.6)	56 (14.0)	5 (1.2)	5 (1.2)	330
**D4. Do you wash your hands after touching unwrapped cooked food?**	310 (77.3)	67 (16.7)	14 (3.5)	10 (2.5)	296
**D5. Do you use separate kitchen utensils to prepare cooked and raw food?**	253 (63.1)	110 (27.4)	33(8.2)	5 (1.3)	220
**D6. Do you thaw frozen food at room temperature?**	246 (61.3)	106 (26.4)	42 (10.5)	7 (1.8)	-204
	TOTAL SCORE/FULL SCORE (%)				1261/2371 (53.2)

### Knowledge

The majority (68.1%) of the respondent nurses agreed that food prepared in advance may contribute to risk of food poisoning, and almost all (91.5%) knew the risk related to reheating dishes prior to consumption (Table 2). Moreover, more than 95% of the nurses agreed that adoption of adequate cleaning and sanitization procedures contributes to control and prevent the risk to consumers. Although almost all nurses believed that wearing gloves while handling food reduce risk of foodborne disease to patients, 61.1% only of the respondents showed awareness of the protective effects of this procedure for food service staff and 30.9% denied these effects. Furthermore, 28.2% of the respondents proved to be unaware of the correct working temperature of a refrigerator and 4.7% did not answer; 83.5% and 37.7%, respectively, did not know the proper storage temperature of hot and cold ready to eat foods. Questions about cholera and gastroenteritis were most frequently answered with an incorrect option or unanswered, but a quite unexpected finding was related to the responses about HBV, where 8.7% only of the nurses included this virus within foodborne agents, but a proportion as high as 51.4% did not answer (Table 2).

Overall, total score of the section "knowledge" for the selected questions accounted for 27.5% of the maximum possible score.

### Attitudes

The most significant responses for this area may be seen in Table 3. Most respondents (78.3%) stated that they intended to separate cooked from raw foods, but 16.0% denied the need of adopting this key measure to prevent cross-contamination, which is a concern. A high proportion of respondents (86.8%) was unaware that defrosted food should not be refrozen. Virtually all respondents (more than 95%) agreed that use of protective clothing and gloves, knowledge and monitoring of refrigerator and freezer temperatures and proper storage of foodstuffs play an important role in preventing food spoilage and health hazard to consumers. Disagreement to the statement that personnel with abrasions or cuts on fingers or hands should not handle unwrapped food was expressed by 12.7% of the respondents.

Overall, total score for the selected questions of the section "attitudes" accounted for 20.0% of the calculated maximum possible score.

### Practices

Responses for this area are displayed in Table 4. An unexpected, but percentually limited, mixed set of responses was evident when considering the practice of washing hands before and after handling unwrapped foods: proportions of respondents ranging from 78.3% to 78.6% for raw foods and from 77.3% to 83.6% for cooked foods stated that they always washed their hands before and after touching food, respectively. Only a little number of respondents stated that they occasionally washed hands while touching unwrapped foods or did not answer. A more heterogeneous set of responses was evident about use of separate utensils for cooked and raw foods: 63.1% only of the respondents fell in the category *always *and 27.4% in the category *often*. Thawing frozen food at room temperature proved to be an extensively used practice, 10.5% only of the nurses stating that they occasionally applied this procedure. Checking shelf life of the products and integrity of packages proved to be a very frequent behaviour within the respondent nurses, more than 95% of them stating that always observed this procedure while buying foodstuffs.

Overall, total score for the selected questions of the section "practices" was 53.2% of the maximum possible score.

### Association between questionnaire answers and demographic and work-related variables

Table 5 shows the results of cross-tabulation and chi-square tests used to identify differences in respondent's knowledge, attitudes and practices about food safety on the basis of gender, age, education, hospital, length of service and previous training on food hygiene. For the purpose of this analysis, knowledge was categorized and compared based upon the criteria described in the statistical procedures (section Materials and Methods). Tables include associations that proved to be statistically significant.

**Table 5:** Selected knowledge, attitudes and practices as influenced by some demographic and work-related characteristics of respondents

**B1. Preparation of food in advance is likely to contribute to food poisoning**			
			
Variable	Correct (%)	Not correct/Don't know (%)	P
Attending courses			
Yes	35 (89.7)	4 (10.3)	0.002*
No	209 (65.5)	110 (34.5)	
			
**B2. Reheating of food is likely to contribute to food contamination**			
			
Variable	Correct (%)	Not correct/Don't know (%)	P

Education level			
8	62 (89.9)	7 (10.1)	
13	231 (92.4)	19 (7.6)	0.026
16	10 (71.4)	4 (28.6)	
			
**B7. The correct temperature for a refrigerator is (°C)**			
			
Variable	Correct (%)	Not correct/Don't know (%)	P

Attending courses			
Yes	32 (86.5)	5 (13.5)	0.038*
No	220 (70.3)	93 (29.7)	
			
**B9. Cold ready to eat food should be maintained at (°C)**			
			
Variable	Correct (%)	Not correct/Don't know (%)	P

Hospital			
General	174 (65.4)	92 (34.6)	0.002*
Paediatric	56 (48.7)	59 (51.3)	
			
Length of service/employment			
<10	50 (64.1)	28 (35.9)	
11–20	98 (66.2)	50 (33.8)	0.012
>20	66 (49.6)	67 (50.4)	
			
Length of service/ward			
<10	62 (62.0)	38 (38.0)	
11–20	84 (62.7)	50 (37.3)	0.054
>20	54 (48.6)	57 (51.4)	
			
**B10b. Hepatitis B can be transmitted by food**			
			
Variable	Correct (%)	Not correct/Don't know (%)	P

Education level			
8	24 (70.6)	10 (29.4)	
13	104 (82.5)	22 (17.5)	0.031
16	2 (40.0)	3 (60.0)	
			
**C1. Raw foods should be kept separated from cooked foods**			
			
Variable	Agree (%)	Don't agree/Uncertain (%)	P

Hospital			
General	234 (84.8)	42 (15.2)	<0.0005*
Paediatric	80 (69.6)	35 (30.4)	
			
**C2. Defrosted food should not be refrozen**			
			
Variable	Agree (%)	Don't agree/Uncertain (%)	P

Hospital			
General	44 (16.1)	230 (83.9)	
Paediatric	1 (0.8)	120 (99.2)	<0.0005
			
Education level			
8	2 (2.9)	66 (97.1)	
13	5 (2.0)	243 (98.0)	0.023
16	2 (14.3)	12 (85.7)	
			
**C7. Food-service staff with abrasions or cuts on hands should not touch unwrapped food**			
			
Variable	Agree (%)	Don't agree/Uncertain (%)	P

Age			
1	74 (93.7)	5 (6.3)	
2	197 (87.2)	29 (12.8)	0.037
3	59 (79.7)	15 (20.3)	
			
Length of service/employment			
<10	70 (90.9)	7 (9.1)	
11–20	136 (89.5)	16 (10.5)	0.040
>20	113 (80.7)	27 (19.3)	
			
**D2. Do you wash your hands after touching unwrapped raw food?**			
			
Variable	Safe (%)	Unsafe (%)	P

Gender			
Male	112 (73.2)	41 (26.8)	0.018*
Female	197 (83.1)	40 (16.9)	
			
**D3. Do you wash your hands before touching unwrapped cooked food?**			
			
Variable	Safe (%)	Unsafe (%)	P

Gender			
Male	122 (79.7)	31 (20.3)	0.045*
Female	206 (87.3)	30 (12.7)	
			
**D5. Do you use separate kitchen utensils to prepare cooked and raw food?**			
			
Variable	Safe (%)	Unsafe (%)	P

Gender			
Male	88 (50.9)	65 (49.1)	0.039
Female	160 (67.8)	76 (32.2)	
			
Education level			
8	50 (75.8)	16 (24.2)	
13	148 (59.4)	101 (40.6)	0.024
16	11 (78.6)	3 (21.4)	
			
**D6. Do you thaw frozen food at room temperature?**			
			
Variable	Safe (%)	Unsafe (%)	P

Hospital			
General	18 (6.6)	255 (93.4)	
Paediatric	24 (19.8)	97 (80.2)	<0.0005*

* P ≤ 0.01 by multivariate logistic regression analysis

### Knowledge

Statistically significant differences were found in respondent's knowledge of food safety issues, when comparing answers to five questions identified in the questionnaire as B1, B2, B7, B9 and B10 b (Table 5). Indeed, nurses of the general hospital "Civico and M. Ascoli" were significantly (*P *= 0.002) more aware than the personnel of the paediatric hospital of the proper temperature of storage of cold ready to eat foods. Persons with an intermediate education level (high school degree) were significantly more likely to know that reheating of food may be a hazardous procedure and to exclude transmission of HBV by food (*P *= 0.026 and *P *= 0.031, respectively). Attending previous courses on food hygiene and foodborne disease was significantly associated (*P *= 0.002) with a higher percentage of correct answers to questions about risks related to preparation of food in advance and safe working temperatures of the refrigerator (*P *= 0.038). Respondents with a higher length of service (≥21 years) were significantly (*P *= 0.012) less likely to indicate the correct temperature of storage of cold foods. Working at the general hospital and having attended courses were significantly associated to a better knowledge by multivariate logistic regression analysis.

### Attitudes

A significantly (*P *< 0.0005) higher proportion of positive attitudes was reported by the nursing staff of the general hospital, 84.8% and 16.1% of whom agreed that raw food should be isolated from cooked food and defrosted food should not be refrozen, respectively, vs. 69.6% and 0.83% of the paediatric hospital's nurses. The issue of refreezing defrosted food was also approached with a significantly safer attitude by respondents with a higher educational level (*P *= 0.023). Respondents with 35 years or less of age and 10 years or less of length of service as a nurse were significantly more likely to pay attention to the need that unwrapped food should not be handled by personnel with cuts or abrasions on their hands (*P *= 0.037 and *P *= 0.040, respectively). Multivariate logistic regression confirmed a more positive attitude toward prevention of cross-contamination in nurses of the general hospital.

### Practices

Consistent with the finding of logistic regression analysis, females were more likely to behave in a safer manner by washing always their hands after touching unwrapped raw food (*P *= 0.018) and before touching unwrapped cooked food (*P *= 0.045). Separating always kitchen utensils for raw and cooked food was more frequently reported by females than males (*P *= 0.039). Education level was also associated with safe activities when handling raw and cooked food by separated utensils (*P *= 0.024): however, respondents with both less and more education were more likely to behave in a safer manner that those with an intermediate degree. Respondents employed in the paediatric hospital were significantly more likely to thaw occasionally food at room temperature (*P *< 0.0005).

### Sources of information on food safety

Proportions as high as 78.1 to 87.7% stated that they had never attended a course on food hygiene and foodborne diseases (Table 1).

From the answers in the section E of the questionnaire, the most prevalent sources of information about food hygiene proved to be mass-media for 41.0% and audio/visual materials for 27.0% of the respondents.

## Discussion

This survey provides information and reveals many critical features about the knowledge, attitudes and practices of nurses occupied as food service staff in two hospitals in Palermo, Italy. Because of the low response rate – 37.1% and 53.5%, respectively, for the general and paediatric hospital – the results could not accurately represent all nurses of the hospitals under study. Moreover, voluntary adherence to filling out the survey questionnaire and self-administration may have select individuals with higher instruction levels and more acute interest toward food safety. Thus, estimates about knowledge, attitudes and practices should to be considered conservative.

Among the respondents, there was a generalized lack of sufficient knowledge of the correct responses about temperature for a safe food storage and etiologic agents and food vehicles associated to some foodborne diseases. The finding that proportions as high as 95% and 43%, respectively, did not know the critical temperature of storage of hot and cold ready to eat foods is of special concern, since nursing staff of the two hospitals under study is routinely involved in the receipt of meals from the external caterer and supervision of their delivery to patients. A similar alarming lack of knowledge about critical temperatures has been reported among food service staff in hospital in Italy and in other countries [19,20]. Nevertheless, the importance of storing foods at correct temperatures has been widely documented and is a basic issue in the implementation of HACCP and in food safety legislation [14,15].

Nevertheless, the survey revealed a general positive attitude toward safe storage practices involving temperature control and correct handling of food using adequate clothing and gloves, but respondents fared worse when they were asked about cross-contamination, refreezing and handling unwrapped food with cuts or abrasions on hands. Comparable results have been obtained from previous studies [19,20].

Self-reported food hygiene behaviours yielded somewhat better results, though some disturbing findings arise from our results: indeed, improper practices, such as sharing of utensils for raw and cooked foods and thawing of frozen food at room temperature, appeared to be widespread among the respondents. Similar behaviours are described in several previous studies and confirm that cross-contamination is a poorly perceived food safety issue [17,19,21,22]. Moreover, washing hands before and after touching unwrapped and raw food was not so generalized as expected in a personnel who should have been continuously trained about hand hygiene. All food service staff, especially in the hospital, should be aware that a careful personal hygiene is a key measure to prevent food contamination and spread of enteric diseases. This is of paramount importance when pathogens have a low minimum infective dose, such E. coli O157 or Norovirus, and their introduction by contaminated food or infected food handlers may be followed by extensive human-to-human transmission [23,24].

Inconsistencies between knowledge, attitudes and practices have been previously detected in previous surveys of food service staff in hospital [19,20]. However, unlike from other Authors, who generally emphasize discrepancy between safe beliefs vs. unsafe practices, in the current study, comparatively better results were obtained for practices than knowledge and attitudes [19,20]. This might be the consequence of lack of specific training, empiric adoption of safe attitudes and behaviours based upon skill in the working and domestic setting, perpetuation of traditional approaches and erratic achievement of information through informal sources.

Contradictory results were obtained when statistically significant associations between some demographic and work-related characteristics and responses to selected questions were analyzed. In a previous study conducted in Italy, younger staff had significantly better knowledge and practices, but this was not true in the present study [19]. Female respondents were more likely than males to wash their hands after touching raw food and before touching cooked food and to separate kitchen utensils. This finding does agree with other surveys on consumer's food safety perception and behaviours, that found risk perception and protective practices more common in the female gender [22]. Education level inconsistently influenced some knowledge, attitudes and practices regarding reheating and refreezing foods, cross-contamination and transmission of HBV, but curiously the 13-year level performed significantly better than the 16-year or more in the section knowledge, though multivariate logistic regression analysis did not support the finding. Overall, surveys have produced inconsistent results with regard to the relation between food safety behaviours and education level, some risky practices being more common within higher education and income level [22].

More than 80% of the respondents did not attend any educational course on food hygiene and foodborne disease. Those who have attended at least one course had a significantly higher knowledge only about risk associated to preparation of food in advance and proper refrigeration temperature. No further differences were detected in both attitudes and practices, suggesting that knowledge alone is probably insufficient to promote positive attitudes and safe behaviours. Similar considerations about the need of alternative educational strategies, such as those based on motivational health education and promotion models, raised from previous studies [19,20].

## Conclusion

Food hygiene in hospital requires special attention to rigorous preventive measures to minimize the hazard of foodborne disease. Several reports document that the concentration of "consumers" at risk may provide a very favourable environment to the diffusion of enteric pathogens from a common source, such as a contaminated meal [10,25]. In this context, the critical role of food handlers has been repeatedly emphasized [7,9]. Of special concern in the nosocomial setting, the possible introduction of pathogens with low minimal infective doses, such as Norovirus, via food and/or an infected food handler, that may be followed by explosive secondary transmission chains with substantial impact on Public Health and economic resources [24].

The hospital food-service system, when contracted out to an external caterer, is considered one of the most complicated production processes within the hospitality sector. Indeed, the diffusion of compulsory competitive tendering, the increasingly demanding, bureaucratic hospital administration regimen, the stringent food costing, the standardization and mass production of meals, the frequent siting of hospitals at considerable distance from the production centre could arise negative effects on both the safety and quality of food. This outlines the need of a strict and systematic monitoring of potential food hazards.

However, in hospital catering, food handlers are very frequently nurses or domestic staff, who are involved in food operations and supervision functions without the preliminary and continuous food safety training and education courses that the European and national legislations mandate for "professional" food handlers. A frequent unawareness of foodborne disease hazards and prevention and control measures is also documented by the present study.

Nevertheless, the implementation of the HACCP system, universally adopted as a proactive method to prevent foodborne disease, does require a team approach and an understanding of the rationale for monitoring procedures by all staff and underscores the need for continuous training. Providing tailored scientifically sound and updated knowledge and identifying factors that could contribute to generate positive attitude and motivate behaviour change in a definite setting could help to minimize foodborne hazard in hospital catering and enhance the practical utility of hygiene training for the personnel involved in food service functions.

## Competing interests

The author(s) declare that they have no competing interests.

## Authors' contributions

CB conceived the study and contributed substantially to acquisition and analysis of data; AC participated in the design of the study and performed the statistical analysis; SG, MG and MLG equally contributed to the analysis and interpretation of data and provided critical review of the manuscript; CM participated in the design and coordination of the study and took the primary role in drafting the manuscript. All authors read and approved the final manuscript.

## Pre-publication history

The pre-publication history for this paper can be accessed here:



## Supplementary Material

Additional file 1 Questionnaire about food safety knowledge, attitudes and practices of nursing staff of two hospitals in Sicily, Italy. It included five sections: a) demographic characteristics, employment status and hospital/ward where the nurse worked; b) knowledge about food hygiene; c) attitudes towards prevention of foodborne diseases; d) measures to be used in prevention of foodborne diseases; e) sources of information about food hygiene. Click here for file

## References

[B1] MacKenzie AA, Allard DG, Perez E, Hathaway S (2004). Food systems and the changing patterns of foodborne zoonoses. Rev Sc Tech.

[B2] Tauxe R (1999). Emerging foodborne diseases: an evolving Public Health challenge. Emerg Infect Dis.

[B3] Buzby JC (2001). Children and microbial foodborne illness. Food Rev.

[B4] Buzby JC (2002). Older adults at risk of complications from microbial foodborne illness. Food Rev.

[B5] Buzby JC, Roberts T (1997). Economic costs and trade impacts of microbial foodborne illness. World Health Stat Q.

[B6] Gerba CI, Rose JB, Haas CN (1996). Sensitive populations: who is at the greatest risk?. Int J Food Microbiol.

[B7] Dryden MS, Keyworth N, Gabb R, Stein K (1994). Asymptomatic food handlers as the source of nosocomial salmonellosis. J Hosp Infect.

[B8] Guallar C, Ariza J, Dominguez MA, Pena C, Grau I, Verdaguer R, Torrens L, Gudiol F (2004). An insidious nosocomial outbreak due to *Salmonella enteritidis*. Infect Control Hosp Epidemiol.

[B9] Maguire H, Pharoah P, Walsh B, Davison C, Barrie D, Threlfall EJ, Chambers S (2000). Hospital outbreak of *Salmonella virchow *possibly associated with a food handler. J Hosp Infect.

[B10] Regan CM, Syedt Q, Tunstall PJ (1995). A hospital outbreak of *Clostridium perfringens *food poisoning – implications for food hygiene review in hospitals. J Hosp Infect.

[B11] Spearing NM, Jensen A, McCall BJ, Neill AS, McCormack GJ (2000). Direct costs associated with a nosocomial outbreak of *Salmonella *infection: an ounce of prevention is worth a pound of cure. Am J Infect Control.

[B12] Barrie D (1996). The provision of food and catering services in hospital. J Hosp Infect.

[B13] Richards J, Parr E, Riseborough P (1993). Hospital food hygiene: the application of Hazard Analysis Critical Control Points to conventional hospital catering. J Hosp Infect.

[B14] Decreto Legislativo n.155 del 26.5.1997 Attuazione delle Direttive 93/43/CE e 96/3/CE concernenti l'igiene dei prodotti alimentari. Gazzetta Ufficiale della Repubblica Italiana n136 del 13061997.

[B15] Regulation (EC) No. 852/2004 of the European Parliament and of the Council on the hygiene of foodstuffs. Official Journal No L 226, 2562004.

[B16] Meakins SM, Adak GK, Lopman BA, O'Brien SJ (2003). General outbreaks of infectious intestinal disease (IID) in hospitals, England and Wales, 1992–2000. J Hosp Infect.

[B17] Angelillo IF, Foresta MR, Scozzafava C, Pavia M (2001). Consumers and foodborne diseases: knowledge, attitudes, and reported behavior in one region of Italy. Int J Food Microbiol.

[B18] Angelillo IF, Viggiani NMA, Rizzo L, Bianco A (2000). Food handlers and foodborne diseases: knowledge, attitudes, and reported behavior in Italy. J Food Prot.

[B19] Angelillo IF, Viggiani NMA, Greco RM, the Collaborative Group (2001). HACCP and food hygiene in hospitals: knowledge, attitudes, and practices of food-services staff in Calabria, Italy. Infect Control Hosp Epidemiol.

[B20] Askarian M, Gholamhosein K, Aminbaig M, Memish ZA, Jafari P (2004). Knowledge, attitudes, and practices of food service staff regarding food hygiene in Shiraz, Iran. Infect Control Hosp Epidemiol.

[B21] Altekruse SF, Yang S, Timbo BB, Angulo FJ (1999). A multi-state survey of consumer food-handling and food-consumption practices. Am J Prev Med.

[B22] Shiferaw B, Cieslak P, Yang S, Angulo F, Vugia D, Marcus R, Koehler J, Deneen V, The FoodNet Working Group (2000). Prevalence of high-risk food consumption and food-handling practices among adults: a multistate survey, 1996–1997. J Food Prot.

[B23] Welinder-Olsson C, Stenqvist K, Badenfors M, Brandberg A, Floren K, Holm M, Holmberg L, Kjellin E, Marild S, Studahl A, Kaijser B (2004). EHEC outbreak among staff at a children's hospital – use of PCR for verocytotoxin detection and PFGE for epidemiological investigation. Epidemiol Infect.

[B24] Zingg W, Colombo C, Jucker T, Bossart W, Ruef C (2005). Impact of an outbreak of norovirus infection on hospital resources. Infect Control Hosp Epidemiol.

[B25] McCall B, McCormack JG, Stafford R, Towner C (1999). An outbreak of *Salmonella typhimurium *at a teaching hospital. Infect Control Hosp Epidemiol.

