# Supplementary material for: Food safety in hospital: knowledge, attitudes and practices of nursing staff of two hospitals in Sicily, Italy
Source: BMC Health Serv Res. 2007 Apr 3;7:45. doi: 10.1186/1472-6963-7-45 (PMC1852552; doi:10.1186/1472-6963-7-45)
Supplement: Additional file 1 — Questionnaire about food safety knowledge, attitudes and practices of nursing staff of two hospitals in Sicily, Italy. It included five sections: a) demographic characteristics, employment status and hospital/ward where the nurse worked; b) knowledge about food hygiene; c) attitudes towards prevention of foodborne diseases; d) measures to be used in prevention of foodborne diseases; e) sources of information about food hygiene. [file 1472-6963-7-45-S1.doc]

Questionnaire

| 1. **Demographic characteristics**   1) Age 2) Sex: M F 3) Ward  Length of service 4) in the employment 5) in the ward  6) Education level  7) Attending of courses on food hygiene and hospital foodborne diseases  **B. Knowledge** The questions below are about knowledge of food preparation and/or preservation, hygiene standards and transmission of foodborne diseases. Please answer by checking *yes, no, I don’t know*.Is preparation of food in advance likely to contribute to food poisoning?Is reheating food likely to contribute to food contamination?Can an incorrect application of cleaning and sanitization procedures for equipment (refrigerator, slicing machine, mincer) increase the risk of foodborne disease to consumers?Can washing hands before handling food reduce the risk of contamination?Can wearing gloves while handling food reduce the risk of transmitting infection to consumers?Can wearing gloves while handling food reduce the risk of transmitting infection to food-services staff?The correct temperature for a refrigerator is: <1°C, 1-5°C, 6-10°C, 11-15°C, 16-20°CHot ready to eat food should be maintained at: 21-30°C, 31-40°C, 41-50°C, 51-60°C, 61-70°CCold ready to eat food (e.g. salami, soft cheese) should be maintained at: <1°C, 1-4°C, 5-8°C, 9-12°C, 13-16°CWhich of the following diseases can be transmitted by food? a) Hepatitis A, b) Hepatitis B, c) Salmonellosis, d) Cholera, e) Botulism, f) GastroenteritisName at least one food item that can be associated with the transmission of the following foodborne diseases: a) Hepatitis A, b) Hepatitis B, c) Salmonellosis, d) Cholera, e) Botulism, f) GastroenteritisC) Attitudes The following statements describe possible attitudes toward food hygiene and related issues. Please indicate your agreement (*yes*) or disagreement (*no*) with each of the following statements or whether you are uncertain about the answer (*uncertain*).  1) Raw food should be kept separated from cooked food  2) Defrosted food should not be refrozen  3) Using cap, masks, protective gloves and adequate clothing reduce the risk of food contamination  4) Is it important to know the temperature of the refrigerator/freezer to reduce the risk of food spoilage  5) Is it necessary to check at regular intervals of time the thermometer setting of refrigerators and freezers  6) Improper storage of foods may be cause of health hazard to consumers  7) Food-services staff with abrasion or cuts on hands should not touch unwrapped food.  **D. Foodborne-Diseases Control Measures**  The questions below refer to measures for foodborne diseases prevention and control. Please answer to each question, by checking *always, often, occasionally* according to your use.  1) Do you wash your hands before touching unwrapped raw food?  2) Do you wash your hands after touching unwrapped raw food?  3) Do you wash your hands before touching unwrapped cooked food?  4) Do you wash your hands after touching unwrapped cooked food?  5) Do you use separate kitchen utensils to prepare raw and cooked food?  6) Do you thaw food at room temperature?  7) Do you check shelf life of food products while buying them? 8) Do you check integrity of food packages while buying food products?E. InformationFrom where do you get information about food hygiene and prevention of foodborne diseases?NowhereEducation courses on food hygieneAudio/visual materialsMass-mediaOther (please specify)……………………………………………………… |
| --- |
